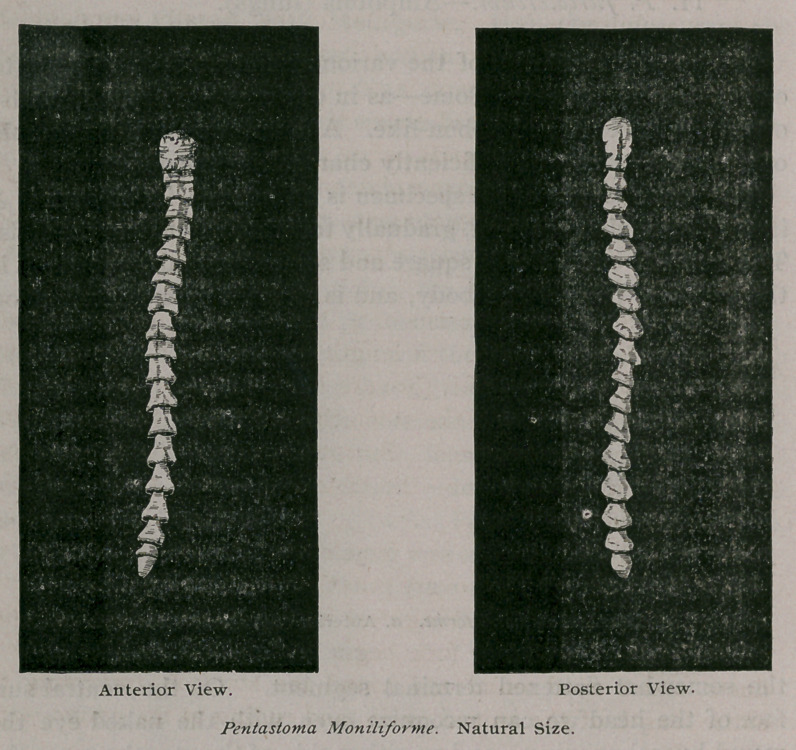# Pentastoma Moniliforme, a Rare Parasite, with Remarks on the Pentastomidæ

**Published:** 1889-01

**Authors:** William S. Gottheil

**Affiliations:** New York


### ART. IV.—PENTASTOMA MONILIFORME, A RARE PARASITE, WITH REMARKS ON THE PENTASTOMIDÆ.

BY WILLIAM S. GOTTHEIL, M. D.

*New York.*

I owe the accompanying parasite to the kindness of Mr. Richardson of the American Museum of Natural History. It was found in the pleural cavity of a large python which had died rather suddenly at the Central Park Menagerie. Half the lung upon the right side had been destroyed, and the parasite had undoubtedly caused the animal's death. The rare occurrence of the entire genus, and especially of this species is such as to warrant a careful examination.

It belongs to the genus Pentastoma, class Arachnida of the Nematode Worms. Whilst some of the genera of the Arachnida are common and well-known parasitic forms—including the Acaridæ (Mites), Ixodidæ (ticks), the Sarcoptes scabaei and the Demodex folliculorum—the Pentastomata are much more uncommon, though they occur throughout the animal kingdom. With the exception of Aves, they have been found in all classes of Vertebrata. They are much commoner in the tropies than in temperate regions, and have been found with especial frequency in tropical America in the lower orders of the Vertebrata, the Reptiles and the Amphibia. The flat Pentastomata are European, whilst the cylindrical ones belong entirely to the New World.

The species of the genus *Pentastoma* (Rud.) are:

A. Single hooks, flat bodies.

1. *P. teniodes.*—Horse, mule, dog, wolf (nostrils).
2. *P. subtriquetrum* (Dies.).—Crocodile (throat).
3. *P. denticulatum* (Rud.).—Goat, guinea-pig, ox, cat (liver, lungs, etc.).
4. *P. serratum* (Rud).—Hare (found in lungs, once).

B. Single hooks, round bodies.

5. *P. oxycephalum* (Dies.).—Crocodile, caiman (in lungs, frequently).
6. *P. subcylindricum.*—Mice, rats, etc. (liver, lungs, etc.).
7. *P. proboscidium* (Rud.).—Rattlesnake and many allied genera (invariably in bronchi and lungs).
8. *P. moniliforme.*—Python, (found once only, and in the lungs).
9. *P. megastomum.*—Lizard (lung).

C. Hooks in pairs, round bodies.

10. *P. gracile.*—Many amphibia and fishes (muscles, intestinal canal, etc.).
11. *P. furcocercum.*—Amphibia (lungs).

The general similarity of the various members of the family to one another is marked. Some—as in our case—are round, whilst others are flattened and ribbon-like. A description of this example of P. moniliforme will sufficiently characterize the genus.

The entire length of the specimen is $2\frac{3}{10}$ inches ; its breadth is $\frac{3}{20}$ inch at the widest segment, gradually tapering slightly to the ends. The cephalic extremity is square and somewhat club-shaped ; it is the broadest part of the body, and is situated immediately upon

Head of *P. moniliforme.* *a.* Anterior *b.* Lateral View.

the somewhat flattened terminal segment. On the ventral surface of the head we can recognize even with the naked eye the round buccal opening ; and on either side of the mouth we see the two lunar slits with convexities outward, which serve as sheaths for the horny and retractile hooks by which the animal fastens itself to the tissues. The number, shape, and position of these hooks varies in the different species. In our specimen there are two on each side, each hook resembling a cat's claw closely in shape. The dorsal surface of the cephalic extremity is smooth and round, and is prolonged over the flat apex as a kind of peaked hood that overhangs the buccal cavity and the hooks.

Below the head commence the ring-like folds, 17 in number, which give to the parasite its peculiar appearance. Save for the fact that each ring is detached from the rest, and runs completely around the body, the general shape would be exactly that of a corkscrew. Alternate rings and interspaces complete the body of the Pentastoma. Each segment increases in size until the lowest one-fourth is reached, after which they rapidly diminish to the caudal extremity.

Anterior View. Posterior View.

*Pentastoma Moniliforme.* Natural Size.

Each ring is umbrella-shaped, and overhangs the succeeding space; measure some $\frac{3}{20}$ inch in diameter, the interspaces measuring half as much only.

The caudal extremity is bluntly truncate and contains the cloaca on its ventral aspect. The oviducts in our specimen open into it.

The cuticle is dirty-white in color, and seems to the naked eye to be quite smooth ; but a low power shows a number of warty prominences covering the upper and lower surfaces, and especially the edges of the segments. Nordemann has held these to be stigmata and believed that they were employed in the absorption of air. We now know that they are only the papillæ that mark the openings of the ducts of the cuticular glands, to be described immediately. In P. moniliforme these papillæ are not so marked as they are in some other varieties; but the integument itself is thick, and does not permit us to see much of the arrangement of the internal organs.

The integument is composed of two chief layers,—of which the outer is a chitinous covering similar to that usually found in the Arachnida. It is an extremely delicate, transparent, and formless membrane. After a layer of large multinucleated cells we come to the corium—composed of connective tissue cells. In this are situated the numerous glands before mentioned.

Each hooklet is fastened to a plate of the same chitinous material that forms the external integument—to each opposite side of which a special muscle is attached. One erects and the other flexes the hook.

The digestive canal consists of a short æsophagus opening into a stomach that runs the entire length of the animal. The oviducts accompany and surround it through its entire course. From the lower and smaller end of the stomach opens the short intestinal canal, running to the cloaca. Surrounding the stomach is a complicated lymphatic plexus—the cisternum chyli, through which absorption occurs.

The specimen before us is a female, and the sexual organs are arranged as follows : the ovary runs the entire length of the animal on the dorsal surface—forking near its anterior end and enclosing the stomach. From the fork begin the extremely complex oviducts, which wind round and round the stomach, traversing the entire length of the animal again, and ending at the genito—anal depression.

The males possess a distinct penis, situated just below the mouth. The testicle begins at the posterior end as a rounded sac, above which lies the epididymis. From the cephalic end of the testicle spring the vasa efferentia, which encircle the anterior portion of the stomach, just as do the horns of the uterus in the female, and end in two small vesiculæ seminale. From the walls of these spring the crura of the penis, the organ itself being covered with ordinary integument. Zoosperms shaped like cercariæ have been seen by Mehlis and Nordemann in P. teniodes.

The nervous system has been found to consist of a large cerebral ganglion which encircles the upper part of the alimentary canal, and from which numerous nerves spread out in various directions. From the lower posterior border of this cerebral ring the nervous system is prolonged into a broad plate, from whose lower borders run two main nerves running the entire length of the animal.

Pentastoma moniliforme is of very rare occurrence, and has been only found in the lung of the python. The single recorded case that I have been able to find is that described by Diesing and obtained by Jul. Czermak from the lung of Python tigris.

## Figures and Tables

**Figure f1:**
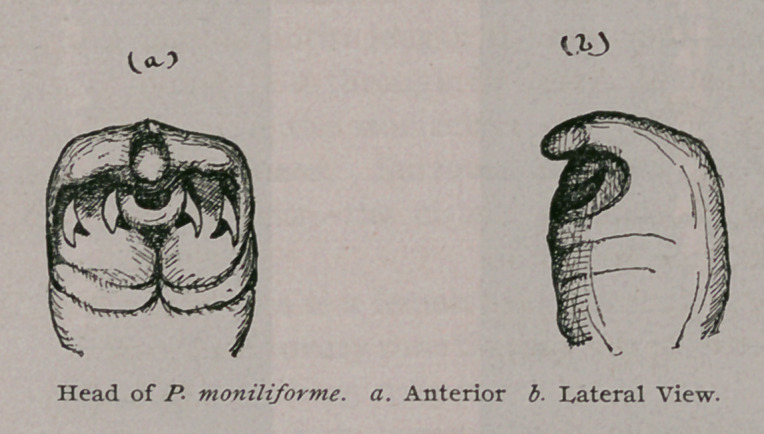


**Figure f2:**